# Genomic and Clinical Features Associated With Reduced Susceptibility to Cefiderocol and Cross-Resistance to β-Lactam/β-Lactamase Inhibitors in a Global Cohort of *Pseudomonas aeruginosa*

**DOI:** 10.1093/infdis/jiag263

**Published:** 2026-05-15

**Authors:** Stephanie L Egge, Donghoon Kang, Nazanin Pouya, Philip C Thornton, Erika Flores, Karim Clarke, Max W Adelman, Cornelius L Rosenbaum, Rodrigo P Baptista, Cesar A Arias, Vincent H Tam, Michael J Satlin, William R Miller

**Affiliations:** Division of Infectious Diseases, Houston Methodist Hospital, Houston, Texas, USA; Center for Infectious Diseases, Houston Methodist Research Institute, Houston, Texas, USA; Division of Infectious Diseases, Houston Methodist Hospital, Houston, Texas, USA; Center for Infectious Diseases, Houston Methodist Research Institute, Houston, Texas, USA; Department of Pharmacy Practice and Translational Research, University of Houston College of Pharmacy, Houston, Texas, USA; Division of Infectious Diseases, Houston Methodist Hospital, Houston, Texas, USA; Center for Infectious Diseases, Houston Methodist Research Institute, Houston, Texas, USA; Division of Infectious Diseases, Houston Methodist Hospital, Houston, Texas, USA; Center for Infectious Diseases, Houston Methodist Research Institute, Houston, Texas, USA; Division of Infectious Diseases, Houston Methodist Hospital, Houston, Texas, USA; Center for Infectious Diseases, Houston Methodist Research Institute, Houston, Texas, USA; Division of Infectious Diseases, Houston Methodist Hospital, Houston, Texas, USA; Center for Infectious Diseases, Houston Methodist Research Institute, Houston, Texas, USA; Department of Medicine, Weill Cornell Medical College, New York, New York, USA; Center of Health Data Science and Analytics, Houston Methodist, Houston, Texas, USA; Division of Infectious Diseases, Houston Methodist Hospital, Houston, Texas, USA; Center for Infectious Diseases, Houston Methodist Research Institute, Houston, Texas, USA; Department of Medicine, Weill Cornell Medical College, New York, New York, USA; Division of Infectious Diseases, Houston Methodist Hospital, Houston, Texas, USA; Center for Infectious Diseases, Houston Methodist Research Institute, Houston, Texas, USA; Department of Medicine, Weill Cornell Medical College, New York, New York, USA; Department of Pharmacy Practice and Translational Research, University of Houston College of Pharmacy, Houston, Texas, USA; Department of Medicine, Weill Cornell Medical College, New York, New York, USA; Division of Infectious Diseases, Houston Methodist Hospital, Houston, Texas, USA; Center for Infectious Diseases, Houston Methodist Research Institute, Houston, Texas, USA; Department of Medicine, Weill Cornell Medical College, New York, New York, USA

**Keywords:** cefiderocol, *Pseudomonas aeruginosa*, antibiotic heteroresistance, ceftolozane/tazobactam, ceftazidime-avibactam

## Abstract

**Background:**

Cefiderocol retains high rates of activity against carbapenem-resistant *P. aeruginosa* (CRPA) and is being introduced as a last resort antibiotic in developing countries. This study assessed the clinical and genotypic characteristics associated with decreased cefiderocol susceptibility among a global collection of CRPA isolates.

**Methods:**

Clinical isolates (n = 186) selected from 972 patients enrolled from 10 countries as part of the Prospective Observational Pseudomonas (POP) study were characterized by whole-genome sequencing, cefiderocol broth microdilution, and population analysis profile. We compared clinical and genomic characteristics between susceptible isolates and those with reduced cefiderocol susceptibility (defined as heteroresistant by population analysis, or intermediate or resistant using current breakpoints). A hollow fiber infection model was used to evaluate the emergence of resistance under human serum simulated cefiderocol exposures.

**Results:**

Of the 186 isolates, 11 (5.9%) were cefiderocol nonsusceptible and 28 (15.1%) were heteroresistant using CLSI breakpoints. With EUCAST breakpoints, 20 (10.8%) were cefiderocol resistant and 20 (10.8%) were heteroresistant. Reduced susceptibility isolates were present across all geographic regions and harbored changes in PirRS-regulated siderophore receptors, AmpC β-lactamase mutations associated with decreased ceftolozane/tazobactam susceptibility, *cpxS* mutations, and the β-lactamases *bla*_NDM_ and *bla*_VEB_. Resistance to ceftolozane/tazobactam and ceftazidime/avibactam were independently associated with reduced cefiderocol susceptibility. Cefiderocol resistance emerged in a heteroresistant, but not susceptible isolate at human simulated exposures.

**Conclusions:**

Decreased cefiderocol susceptibility is present worldwide and is not restricted to a clonal high-risk lineage. This raises concerns about the use of cefiderocol as a salvage regimen in patients with multidrug-resistant *P. aeruginosa* infection.

## Teaser

Cefiderocol heteroresistance was present globally in an international cohort of carbapenem-resistant *Pseudomonas aeruginosa*, associated with acquired β-lactamases, changes in AmpC, and iron import pathways. Ceftolozane/tazobactam and ceftazidime-avibactam resistance were associated with decreased cefiderocol susceptibility, suggesting caution with cefiderocol salvage therapy.

*Pseudomonas aeruginosa* is a leading cause of healthcare-associated infections, particularly in critically ill patients and those with chronic health conditions such as cystic fibrosis [1]. Carbapenem-resistant *P. aeruginosa* (CRPA) causes substantial mortality worldwide and is designated a high-priority pathogen by the World Health Organization [2, 3]. Treatment options are limited, as CRPA isolates acquire β-lactam resistance via multiple mechanisms, including porin loss, changes in penicillin-binding proteins (PBPs), increased hydrolytic activity of the intrinsic pseudomonal-derived cephalosporinase (AmpC), and acquired β-lactamases [4]. Cefiderocol stands as a last-line therapy for CRPA, due to in vitro activity against extensively drug-resistant and metallo-β-lactamase-producing isolates and is prioritized for distribution in up to 135 low and middle-income countries across Latin America, Africa, and Asia [5, 6]. However, concerns have arisen over difficulties with susceptibility testing, reports of clinical failures, and the emergence of cefiderocol resistance [7-10].

Cefiderocol binds iron, facilitating uptake by specialized siderophore channels called TonB-dependent receptors [11]. Changes in the TonB-dependent receptors PirA, PiuA, the regulatory proteins PirRS or PiuC, and loss of the ferripyochelin receptor FptA reduce antibiotic entry, analogous to porin channel loss for other β-lactams [12, 13]. Further, mutations in the intrinsic AmpC and presence of NDM β-lactamases have been linked to elevated cefiderocol minimum inhibitory concentrations (MIC) [14, 15]. Additionally, CpxS (which upregulates the MexAB-OprM/MuxABC-OpmB efflux pumps) and PBP3 changes have also been associated with reduced cefiderocol activity [9, 16, 17]. However, there is uncertainty about the prevalence and impact of these mechanisms in clinical strains worldwide [18, 19].

In addition to classically defined resistance, *P. aeruginosa* may display a heteroresistance phenotype, often even before cefiderocol exposure [9, 10, 20]. Heteroresistant isolates exhibit minority subpopulations with decreased antimicrobial susceptibility, despite testing susceptible via reference broth microdilution (BMD) [21]. Limited data suggest that genotypic determinants of cefiderocol heteroresistance overlap with traditional resistance mechanisms, however, the prevalence and potential mechanisms behind this phenotype remain incompletely understood [22]. Using an international collection of CRPA isolates collected as part of the Prospective Observational Pseudomonas (POP) study [23], we sought to determine the global distribution of decreased susceptibility to cefiderocol, identify clinical characteristics and molecular determinants associated with loss of cefiderocol susceptibility, evaluate cross-resistance to newer β-lactam/β-lactamase inhibitors, and determine whether human simulated exposures of cefiderocol suppress the emergence of cefiderocol resistance in heteroresistant isolates.

## METHODS

### Genomic Analysis of *P. aeruginosa* Isolates

The POP study was conducted by the Antibiotic Resistance Leadership Group (ARLG) and enrolled 972 patients globally from December 2018 to November 2019 (prior to the clinical introduction of cefiderocol), with first isolated CRPA strain (meropenem MIC ≥8 μg/mL), susceptibility testing data, whole-genome sequences, and de-identified clinical data maintained in a biorepository [23]. Ethics approval for the current study was waived after review by the Houston Methodist Institutional Review Board (PRO00037122) as this was secondary use of previously collected isolates and de-identified data. Genomic analysis (see Supplementary Methods) included screening for mutations in TonB-dependent receptor genes, *ampC* variants, and the presence of β-lactamases. Briefly, maximum likelihood trees for each gene were constructed using RAxML-NG (version 1.2.0) to group the different gene alleles, with the 972 isolates containing 41 variants of *pirR*, 61 variants of *pirS*, 53 variants of *piuA*, 29 variants of *piuC*, and 47 variants of *ampC* identified [24]. Isolates were then selected at random from these groups. Of the original selection, a minority of strains (10 of 196 isolates) clustered on a distinct phylogenetic branch consistent with *P. paraeruginosa* (PA-7 type strain, approximately 93.5% average nucleotide identity [ANI] to *P. aeruginosa* sensu stricto) [25]. These strains were excluded from analysis due to the ANI divergence. The final phenotypic cohort (n = 186) was acquired from the ARLG isolate bank for phenotypic characterization.

### Antimicrobial Susceptibility Testing

Mueller-Hinton (MH) II broth (BBL Becton Dickinson, Fisher Scientific) was iron-depleted (ID-MH) according to Clinical and Laboratory Standards Institute (CLSI) guidance for cefiderocol (Shionogi, Inc.) BMD susceptibility testing [26]. Cefiderocol susceptibility testing was performed in triplicate on independent days. Kirby-Bauer disk diffusion was performed using 30 µg cefiderocol disks (Hardy Diagnostics) on MH agar (BBL Becton Dickinson, Fisher Scientific). Disk diameters were recorded at 24 and 48 hours, based on highest proximity single colony growth relative to the disk. Fisher's exact test was used to compare disk diffusion zone diameters and MIC distributions across these groups. Antimicrobial susceptibility testing by agar dilution for ceftolozane/tazobactam and ceftazidime-avibactam, and BMD for imipenem-relebactam were previously performed at the ARLG central laboratory. Cefiderocol BMD susceptibility was interpreted using both CLSI and European Committee on Antimicrobial Susceptibility Testing (EUCAST) breakpoints.

### Assessment of heteroresistance by population analysis profile (PAP)

Heteroresistance was assessed by PAP as previously described [22]. Strains were grown overnight in ID-MH broth, standardized to 0.5 McFarland, with serial dilutions plated on MH agar containing cefiderocol at concentrations of 0, 4, 8, and 16 μg/mL. Colony forming units (CFU) were counted at 24 and 48 hours. Log-normalized CFU changes at each cefiderocol concentration were calculated relative to the growth control. The trapezoidal method was used to calculate area under the curve (AUC) in Prism (GraphPad), with heteroresistance assessed by both CFU criteria (<50% survival at 4 µg/mL with >0.0001% survival at 8 µg/mL, compared with growth without antibiotic) and AUC >80 [22, 27]. Isolates that screened negative on PAP (>6 log_10_ decrease in CFU/mL compared with no antibiotic control) and had a susceptible BMD MIC were assayed once and considered fully susceptible. Population analysis profile was repeated in triplicate on different days for isolates with <6 log_10_ decrease in CFU/mL or BMD MIC of ≥8 µg/mL to confirm the phenotype.

### Hollow Fiber Infection Model

A hollow fiber infection model (HFIM) was used to simulate the human serum exposures of 2 g cefiderocol given over 3 hours every 8 hours against a previously published *P. aeruginosa* strain pair [9]. The strain pair was utilized to control for strain background, as no isolate pairs were available in the POP isolate collection. The susceptible strain BSL5 (cefiderocol MIC 2 µg/mL, AUC 45.4) possesses *bla*_VIM-4_ but no mutations in cefiderocol target genes. The heteroresistant strain BSL9 (cefiderocol MIC 4 µg/mL, AUC 90.6) also possesses *bla*_VIM-4_ and additionally a premature stop codon in the *fptA* gene. The target unbound Cmax was 60 mg/L, half-life 2.5 hours, confirmed by LC-MS/MS [28]. Tryptic soy broth supplemented with deferiprone (15 μg/mL) was used as diluent [29]. A baseline inoculum of approximately 10^8^ CFU/mL was used. Change in bacterial density was monitored for up to 72 hours by colony counts on MH agar and MH agar with cefiderocol at 3×MIC to assess resistance emergence.

### Statistical Analysis

POP cohort clinical characteristics were expressed as the median and range for continuous variables, and frequency and percentage for categorical data. Antimicrobial susceptibility for the cohort analysis was determined using CLSI breakpoints. Descriptive statistics for the combined cefiderocol heteroresistant and nonsusceptible population versus the cefiderocol susceptible population were calculated, with a significance threshold of *P* < .05. Differences in continuous variables were determined using a 2-sample *t* test with equal variance. Differences in categorical variables were determined using *χ*² test. Fisher's exact categorical analyses were used to assess associations between cefiderocol susceptibility phenotype and target genes. Odds ratio and associated 95% CIs were calculated assessing odds of gene presence among cefiderocol heteroresistant plus nonsusceptible versus susceptible strains.

Overall prevalence of resistance to ceftolozane-tazobactam, ceftazidime-avibactam, and imipenem-relebactam between cefiderocol susceptible and heteroresistant/nonsusceptible isolates was compared using *χ*² test. We determined odds of cefiderocol heteroresistance or nonsusceptibility according to ceftolozane-tazobactam, ceftazidime-avibactam, and imipenem-relebactam resistance (as well as combinations of these drugs) using univariable logistic regression models. Logistic regression was also used to assess overall association of the cefiderocol heteroresistant or nonsusceptible phenotype with total number of resistant β-lactam/β-lactamase inhibitors (BL/BLIs). We then constructed multivariable logistic regression models adjusting for nonsusceptibility to other BL/BLIs to assess the independent association of resistance to each drug with cefiderocol resistance. Data were analyzed with SAS Studio (SAS Institute, Cary, NC).

## RESULTS

### Clinical Features and Geographic Distribution of Reduced Cefiderocol Susceptibility

Among the 186 representative POP isolates, 11 (5.9%) displayed cefiderocol MIC above the CLSI susceptible breakpoint of 4 μg/mL (nonsusceptible population, Figure 1*A*). Twenty-eight (15.1%) isolates were classified as heteroresistant, defined by a PAP-area under the curve (PAP-AUC) value of >80 [22], and 19 isolates met the CFU definition of heteroresistance defined by a less than 6 log_10_ drop in colony-forming units (CFU) per mL at the clinically-defined antimicrobial resistance breakpoint (Supplementary Table 1, Supplementary Figure 1) [30]. Using the EUCAST susceptibility breakpoint of 2 μg/mL, 20 (10.8%) isolates were resistant and 20 (10.8%) were heteroresistant. Nine isolates had an MIC of 4 μg/mL (ie, considered resistant by EUCAST but susceptible by CLSI breakpoints) and of these 8 were heteroresistant by both PAP-AUC and CFU criteria. Of the AUC-defined heteroresistant isolates, disk zone diameters were associated with susceptibility phenotypes (Figure 1*B*). There was a clear difference between the mean disk diffusion diameter of both heteroresistant (Δ diameter −5.5 mm, *P* < .0001) and nonsusceptible (Δ diameter −7 mm, *P* < .0001) isolates as compared with the fully susceptible population (mean 29 mm).

**Figure 1.**
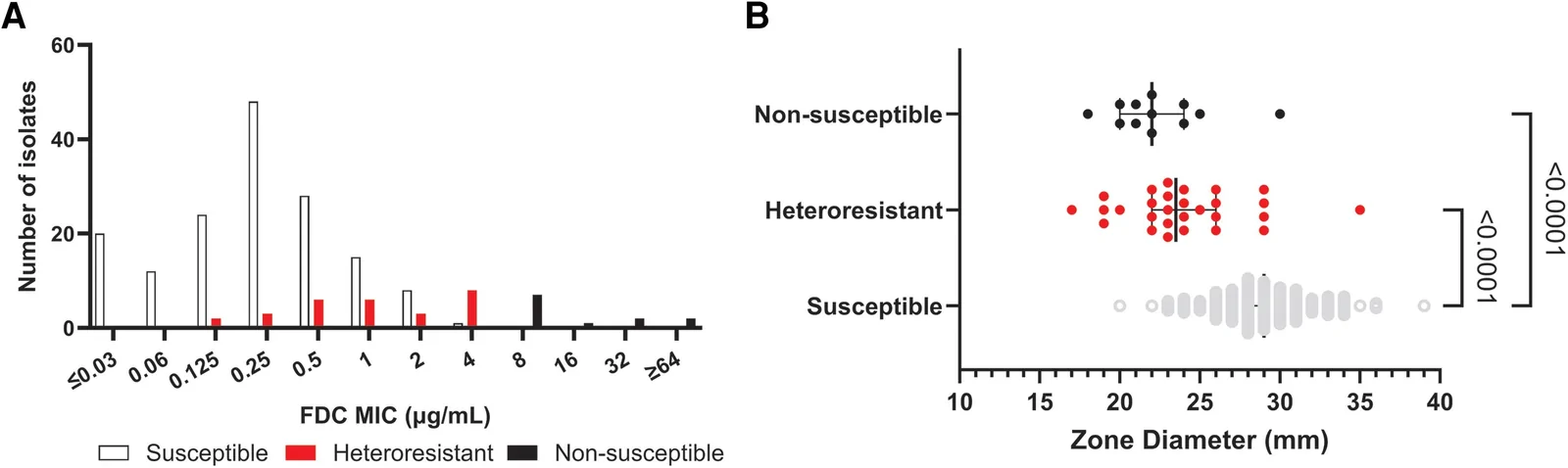
Cefiderocol minimum inhibitory concentration and disk diffusion diameter distribution among POP isolates according to cefiderocol susceptibility phenotype. *A*, Minimum inhibitory concentration and *B*, disk diffusion diameter distribution among POP isolates according to cefiderocol susceptibility phenotype. For disk diameters, error bars show the median and 95% CI. *P* values are noted for the Mann–Whitney test comparing differences in the median diameter of susceptible isolates to heteroresistant or nonsusceptible isolates, respectively. Cefiderocol nonsusceptible strains based on broth microdilution, intermediate (8 µg/mL) or resistant (≥16 µg/mL) by CLSI interpretations. *P* values are shown for each comparison. Abbreviations: FDC, cefiderocol; MIC, minimum inhibitory concentration.

Next, we analyzed the POP cohort clinical data stratified by cefiderocol heteroresistant or broth microdilution nonsusceptible (reduced cefiderocol susceptibility, n = 39) and susceptible (n = 147) groupings based on CLSI breakpoints. Evaluation of clinical features revealed no significant differences in patient demographics, patient comorbidities, time from hospital admission to positive culture, isolate anatomic source, community versus healthcare onset, and whether the cultured isolate was considered colonization versus infection (Table 1). Immunocompromised status was more common among patients with susceptible isolates versus those with reduced cefiderocol susceptibility (15% vs 2.6%, *P* = .051). Isolates with reduced cefiderocol susceptibility were distributed across geographic regions (*P* = .04), with US isolates representing 56% of these strains. The remaining isolates originated from Middle East (20%), Asia (7.5%), Latin America (7.7%), and Oceania (7.5%). The clinical features of the phenotypic cohort were similar to the remaining 786 isolates of the POP study, with the exception of geographic location (since isolates from China were not available in the biorepository) and a larger number of wound isolates in the phenotypic cohort (24% vs 15%, *P* = .027; Supplementary Table 2).

**Table 1.** Clinical Features of Patients Harboring Cefiderocol Susceptible vs Cefiderocol Heteroresistant or Nonsusceptible *P. aeruginosa* Isolates

	Cefiderocol Susceptible, N = 147^a^	Cefiderocol hR + NS, N = 39^a^	*P* Value^b^
Patient demographics	…	…	…
Age, at time of culture	58 (39–71)	63 (49–73)	.30
Gender (male: female)	87 (59%) : 60 (41%)	24 (62%) : 15 (38%)	.80
Geographic location	…	…	**.04**
Asia	2 (1.9%)	3 (7.5%)	…
Oceania	2 (1.9%)	3 (7.5%)	…
Middle East	21 (14%)	8 (20%)	…
North America	87 (59%)	22 (56%)	…
South/Central America	35 (22%)	3 (7.7%)	…
Patient comorbidities	…	…	…
Charlson Comorbidity Score	2 (0–3)	2 (1–4)	.30
Chronic obstructive pulmonary disease	20 (14%)	3 (7.7%)	.40
Cardiac disease	36 (24%)	10 (26%)	.90
Cerebrovascular disease	25 (17%)	7 (18%)	>.9
Diabetes	40 (27%)	15 (36%)	.40
Liver disease	11(7.5%)	0 (0%)	.12
Chronic kidney disease	23 (16%)	8 (21%)	.40
Hemodialysis or peritoneal dialysis	15 (10%)	6 (15.0%)	.11
Immunocompromised state	22 (15%)	1 (2.6%)	.051
Connective tissue disease	6 (4.2%)	0 (0%)	.30
Hematopoietic stem cell transplant	2 (1.3%)	0 (0%)	N/A
Solid organ transplant	9 (6.2%)	0 (0%)	.80
Heart	2 (1.4%)	0 (0%)	…
Kidney	5 (3.4%)	0 (0%)	…
Lung	2 (1.4%)	0 (0%)	…
Isolate features	…	…	…
Days from admission to culture	11 (1–32)	13 (0–28)	.50
Patient location at first culture	…	…	.40
Emergency department	18 (12%)	7 (18%)	…
Medical ward	46 (31%)	14 (36%)	…
Hematology/oncology ward	4 (2.7%)	2 (5.1%)	…
Surgery ward	6 (4.1%)	3 (7.7%)	…
Intensive care unit	64 (44%)	12 (31%)	…
Other	9 (6.1%)	1 (2.6%)	…
Source	…	…	.60
Blood	12 (8.2%)	3 (7.7%)	…
Respiratory	73 (50.0%)	15 (38%)	…
Urine	28 (19%)	10 (26%)	…
Wound	34 (23%)	11 (28%)	…
Polymicrobial culture	44 (30%)	17 (44%)	.11
Community v. healthcare onset	85 (58%) v. 62 (42%)	22 (56%) v. 17 (44%)	.60
infection (not colonization)	78 (53%)	23 (59%)	.50

Bolded values are *P* < .05.

Abbreviations: hR, heteroresistant; NS, cefiderocol nonsusceptible with iron-depleted Mueller-Hinton broth microdilution MIC ≥8 µg/mL.

^a^Median (Q1, Q3); n (%).

^b^Wilcoxon rank sum test; Pearson's *χ*² test; Fisher's exact test.

### Genomic Features Associated With Reduced Cefiderocol Susceptibility

Using whole-genome sequencing, we characterized the phylogenetic distribution and resistance determinants present in isolates according to cefiderocol susceptibility phenotype (Figure 2*A*). Isolates with reduced susceptibility were distributed across the cohort and did not cluster to a specific clade or high-risk clone (Supplementary Figure 2). Overall, the presence of an acquired β-lactamase was more common in isolates from outside of the USA, while PBP3 changes, *ampC* mutations, and *pirRS* mutations were associated with US isolates (Figure 2*B*). Acquired β-lactamase enzymes demonstrated a variable correlation with cefiderocol susceptibility phenotype (Table 2). All isolates producing NDM (n = 4, all *bla*_NDM-1_) were either heteroresistant by PAP (n = 3) or resistant (n = 1) by BMD, and originated from the USA (1), Australia (1), and Asia (2). All 5 isolates producing *bla*_VEB_ were either heteroresistant (n = 4, 3 *bla*_VEB-9_ and 1 *bla*_VEB-16_) or resistant (n = 1, *bla*_VEB-9_). Four of 5 VEB-containing isolates originated from the Middle East, and the other was isolated in the USA. The presence of *bla*_VIM_, *bla*_IMP_, *bla*_KPC_, and *bla*_CTX-M_ were not associated with decreased cefiderocol susceptibility.

**Figure 2.**
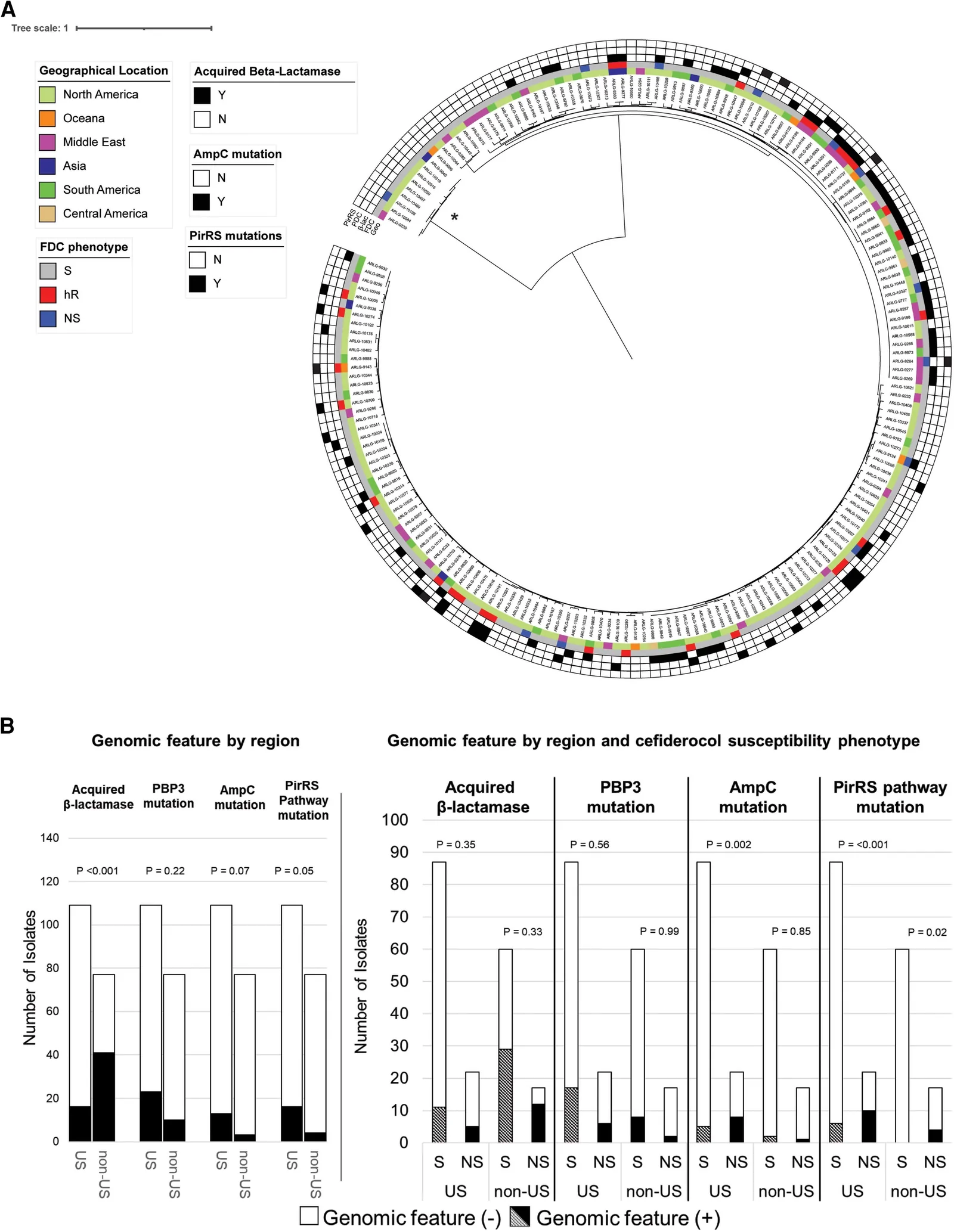
Maximum likelihood phylogenetic tree and geographic associations of the phenotypic cohort. *A*, Maximum likelihood phylogenetic tree of the phenotypic cohort of *P. aeruginosa* isolates. Ten (10/196, 4.6%) clustered on a distinct branch, denoted by (*), consistent with the proposed species *P. paraeruginosa* (type strain PA-7, average nucleotide identity [ANI] of approximately 93.5% as compared with *P. aeruginosa* sensu stricto) and were excluded from further analysis. From inside to outside, rings denote geographic region of isolation, cefiderocol (FDC) phenotype, presence of an acquired β-lactamase, changes in AmpC predicted to alter ceftolozane hydrolysis (F121L, ΔR212-L218, V213A, G216D, P217L, E221K, E221G, A293P, ΔA293-L294, L294P, Q295R), and major mutations in genes of the *pirRS* pathway. *B*, Distribution of genomic features by geographic region and cefiderocol susceptibility phenotype. *P* values for the association of genotypic feature with geographic grouping or phenotype are listed above each comparison. Abbreviations: FDC, cefiderocol; MIC, minimum inhibitory concentration; NS, nonsusceptible; PBP, penicillin-binding protein; S, susceptible; US, United States.

**Table 2.** Genotypic Features Associated With Cefiderocol Susceptible, Heteroresistant, and Nonsusceptible Phenotypes

Genotypic Feature	Susceptible (n = 147)	Not Susceptible (n = 39)		Odds Ratio of Not Susceptible Phenotype Given Genotypic Feature, (95% CI)	*P* Value S V. hR + MIC ≥8 µg/mL
	…	Heteroresistant (n = 28)	MIC ≥8 µg/mL (n = 11)		
TonB-dependent receptor pathway^a^	…	…	…	…	…
Major mutations in PirRS pathway components^b^	7 (4.8)	11 (39.3)	3 (27.3)	11.2 (4.22 to 28.02)	**<**.**0001**
Major mutations in other TBDR	43 (29.3)	11 (39.3)	2 (18.2)	1.21 (.58 to 2.62)	0.6952
FemA	14 (9.5)	4 (14.3)	1 (9.1)	1.40 (.52 to 4.10)	0.5558
FptA	1 (0.7)	0 (N/A)	0 (N/A)	0 (0 to 33.92)	>.9999
OptE	25 (17.0)	7 (25.0)	1 (9.1)	1.26 (.54 to 2.90)	.6393
PfuA	6 (4.1)	1 (3.6)	0 (N/A)	0.62 (.05 to 4.00)	>.9999
TonB Complex	30 (20.4)	9 (32.1)	3 (27.3)	1.56 (.64 to 3.80)	.3276
TonB1	2 (1.4)	0 (N/A)	0 (N/A)	0 to 11.04	>.9999
TonB2	28 (19.0)	8 (20.5)	3 (27.3)	1.75 (.77 to 3.72)	.2676
ExbB1	0 (N/A)	1 (3.6)	0 (N/A)	∞ (.42 to ∞)	.2097
Other intrinsic mechanisms	…	…	…	…	…
AmpC changes predicted to alter C/T activity^c^	7 (4.8)	8 (28.6)	1 (9.1)	6.00 (2.11 to 15.64)	.**0012**
Mutations in *ftsI* (PBP3)	25 (17.0)	4 (14.3)	4 (36.3)	1.26 (.54 to 2.90)	.6393
Major mutations in *cpxS*^b^	0 (N/A)	4 (14.3)	0 (N/A)	∞ (3.88 to +∞)	**.0017**
Acquired β-lactamases	…	…	…	…	…
Presence of any acquired β-lactamase	40 (27.2)	12 (42.9)	5 (45.5)	2.07 (1.00 to 4.14)	.0535
Presence of any metallo-β-lactamase	12 (8.2)	5 (17.9)	1 (9.1)	2.05 (.71 to 5.94)	.2199
Presence of VIM	10 (6.8)	1 (3.6)	0 (N/A)	0.36 (.03 to 2.25)	.4632
Presence of NDM	0 (N/A)	3 (10.7)	1 (9.1)	∞ (3.88 to +∞)	.**0017**
Presence of IMP	4 (2.7)	1 (3.6)	0 (N/A)	0.94 (.08 to 5.96)	>.9999
Presence of any serine carbapenemase	14 (9.5)	1 (3.6)	1 (9.1)	0.51 (.11 to 2.11)	.5298
Presence of KPC	11 (7.5)	1 (3.6)	1 (9.1)	0.67 (.14 to 3.00)	>.9999
Presence of any ESBL	8 (5.5)	4 (14.3)	4 (36.3)	4.45 (1.49 to 13.22)	.**0069**
Presence of CTX-M	4 (2.8)	0 (N/A)	1 (9.1)	0.95 (.08 to 5.96)	>.9999
Presence of VEB	0 (N/A)	4 (14.3)	1 (9.1)	∞ (5.96 to ∞)	.**0003**
Mutation combinations	…	…	…	…	…
AmpC mutants lacking PirRS pathway mutations	7 (4.8)	3 (11.1)	1 (9.1)	2.29 (.71 to 7.79)	.2465
PirRS pathway mutants lacking AmpC mutations	7 (4.8)	6 (21.4)	3 (27.3)	6.00 (2.11 to 15.64)	.**0012**
AmpC plus PirRS pathway mutations	0 (N/A)	5 (17.9)	0 (N/A)	∞ (5.96 to +∞)	.**0003**

Bolded values are *P* < .05.

Abbreviations: hR, heteroresistant; NS, cefiderocol nonsusceptible with iron-depleted Mueller-Hinton broth microdilution MIC ≥8 µg/mL; PBP, penicillin-binding protein.

^a^fepA and exbD1 did not show major mutations in any isolate and are not listed in the table.

^b^Stop codon, frameshift, mutations leading to codon insertion/deletion, or full gene deletion.

^c^AmpC changes predicted to alter activity of ceftolozane/tazobactam [C/T]: F121L, ΔR212-L218, V213A, G216D, P217L, E221K, E221G, A293P, ΔA293-L294, L294P, Q295R.

Analysis of intrinsic genes included siderophore uptake pathways (outer membrane TonB-dependent receptors and inner membrane proteins that make up the TonB complex) and genes that are not directly related to siderophore import, including *ampC*, *ftsI*, and *cpxS* (Table 2). Isolates with probable loss of function mutations (eg, insertion, deletion, early stop codon, frameshift, or gene deletion) in the PirRS-PirA and PiuA/D TonB-dependent receptor pathway components were more likely to display reduced cefiderocol susceptibility as compared with susceptible isolates (OR 11.2, 95% CI 4.22 to 28.02). There was no difference in the mutation prevalence in the genes encoding the TonB-dependent receptors FemA, FepA, FptA, OptE, or PfuA across the susceptible and reduced susceptibility isolates, and no differences in the genes encoding the inner membrane TonB complex. Mutations in *ampC* leading to alterations of the Ω-loop and YSN-loop were associated with increased likelihood of reduced cefiderocol susceptibility (OR 6.00, 95% CI 2.11 to 15.64). All isolates with major mutations in *cpxS* (n = 4) were found to have reduced cefiderocol susceptibility. No notable differences in PBP3 were found between the 2 groups.

We performed a gene copy number analysis among isolates with acquired β-lactamases (Supplementary Figure 3), since copy number variation (CNV) has been linked to heteroresistance in Enterobacterales [31-33]. Estimated CNV of acquired β-lactamase genes did not seem to follow a pattern of increased copy number in the heteroresistant isolates as compared with the susceptible isolates.

Strains with mutations in both the PirRS and AmpC pathways were more likely to have a reduced cefiderocol susceptibility than isolates with mutations exclusively in 1 pathway. Strains with isolated *ampC* mutations showed no statistically significant difference in prevalence among cefiderocol susceptible versus reduced susceptibility isolates. Isolates with changes in the PirRS pathway alone were more likely to have decreased cefiderocol susceptibility (OR: 6.00, 95% CI 2.11 to 15.64), while all isolates (n = 5) harboring changes in both AmpC and PirRS pathways exhibited a heteroresistant phenotype.

### Resistance to Newer β-Lactam/β-Lactamase Inhibitor Anti-pseudomonal Drugs is Associated With Decreased Cefiderocol Susceptibility

Since cefiderocol is often employed as a salvage regimen for isolates resistant to other β-lactams or BL/BLIs, we assessed the association of pre-existing ceftolozane/tazobactam, ceftazidime-avibactam, and imipenem-relebactam resistance with risk for reduced cefiderocol susceptibility (Table 3, Supplementary Figure 4). Isolates with ceftolozane/tazobactam MIC > 4/4 μg/mL were significantly more likely to have a nonsusceptible or heteroresistant phenotype (OR 6.45, 95% CI 2.89–13.99). Isolates with ceftazidime-avibactam MIC > 8/4 μg/mL also showed a significant increase in the probability of a nonsusceptible or heteroresistant phenotype (OR 8.06, 95% CI 3.53–18.40), with similar findings for isolates with imipenem-relebactam MIC > 2/4 μg/mL (OR 2.90, 95% CI 1.20–7.00).

**Table 3.** Reduced Cefiderocol Susceptibility According to β-lactam/β-lactamase Inhibitor Resistance Phenotype

	Cefiderocol Susceptible, N = 147 N(%)	Cefiderocol Nonsusceptible, N = 39 N(%)	Odds Ratio of FDC Resistance (95% CI)	*P* Value
C/T MIC > 4 μg/mL	38 (25.9)	27 (69.2)	6.45 (2.98–13.99)	<.0001
CZA MIC > 8 μg/mL	43 (29.3)	30 (76.9)	8.06 (3.53–18.40)	<.0001
IMR MIC > 2 μg/mL	90 (61.2)	32 (82.1)	2.90 (1.20–7.00)	<.0001
Logistic regression by cumulative BL/BLI resistance				
No novel BL/BLI resistance	50 (34.0)	2 (5.1)	Referent	-
Resistance to 1 novel BL/BLI	49 (33.3)	5 (12.8)	2.55 (.47–13.77)	.28
Resistance to 2 novel BL/BLIs	22 (15.0)	12 (30.8)	13.63 (2.81–66.08)	.001
Resistance to all 3 novel BL/BLIs	26 (17.7)	20 (51.3)	19.22 (4.17–88.66)	.0002
Multivariate logistic regression assessing independent BL/BLI association with cefiderocol nonsusceptibility				
Odds Ratio cefiderocol nonsusceptibility independently associated C/T resistance (95% CI)	…	…	2.61 (1.01–6.75)	.05
Odds Ratio cefiderocol nonsusceptibility independently associated CZA resistance (95% CI)	…	…	4.23 (1.53–11.65)	.005
Odds Ratio cefiderocol nonsusceptibility independently associated IMR resistance (95% CI)	…	…	1.29 (.47–3.52)	.62

Abbreviations: BL/BLI, β-lactam/β-lactamase inhibitor; C/T, ceftolozane/tazobactam; CZA, ceftazidime-avibactam; FDC, cefiderocol; IMR, imipenem-relebactam.

Using logistic regression, we compared the odds of having a heteroresistant or nonsusceptible phenotype as a function of cumulative resistance to the newer BL/BLIs. Decreased susceptibility to cefiderocol was significantly associated with resistance to 2 novel BL/BLI agents (OR 13.63, 95% CI 2.81–66.08) or all 3 agents (OR 19.22, 95% CI 4.17–88.66). A multivariate analysis to assess the contribution of each individual agent showed decreased cefiderocol susceptibility was primarily driven by isolates with resistance to ceftolozane-tazobactam (OR 2.61, 95% CI 1.01–6.75) and ceftazidime-avibactam (OR 4.23, 95% CI 1.53–11.65).

### Heteroresistance is Associated With Treatment Failure at Human Simulated Cefiderocol Exposures in a Hollow Fiber Infection Model.

We utilized a hollow fiber model to evaluate the activity of cefiderocol and the propensity for the emergence of resistance between a susceptible (BSL5) and heteroresistant (BSL9) isolate pair at human serum antibiotic exposures [9]. Pharmacokinetic simulations in the hollow fiber model were satisfactory (*r*^2^ > 0.98, Supplementary Figure 5). After 72 hours of exposure, there was a 3-log_10_ decrease in CFU/mL of the susceptible BSL5 strain, with the suppression of any colonies growing on the resistance screening plates at cefiderocol concentrations of 6 µg/mL or greater (Figure 3*A*). In contrast, the heteroresistant isolate BSL9 showed only a 1.3-log_10_ decrease in CFU/mL at 72 hours, with 17% of the population (5.6 log_10_ CFU/mL) growing at cefiderocol concentrations of 12 µg/mL or greater (Figure 3*B*). An isolate selected at random from the resistant population underwent cefiderocol BMD with an MIC of 128 µg/mL (disk diameter 13 mm), confirming the emergence of resistance. This may occur via selection of a population that transiently displays a higher MIC (unstable heteroresistance) or by selection of additional mutations resulting in a fixed resistance phenotype. After passage in antibiotic free media for 72 hours, the isolate maintained a disk diameter of 13–14 mm, suggesting selection of a fixed resistance phenotype from the heteroresistant subpopulation.

**Figure 3.**
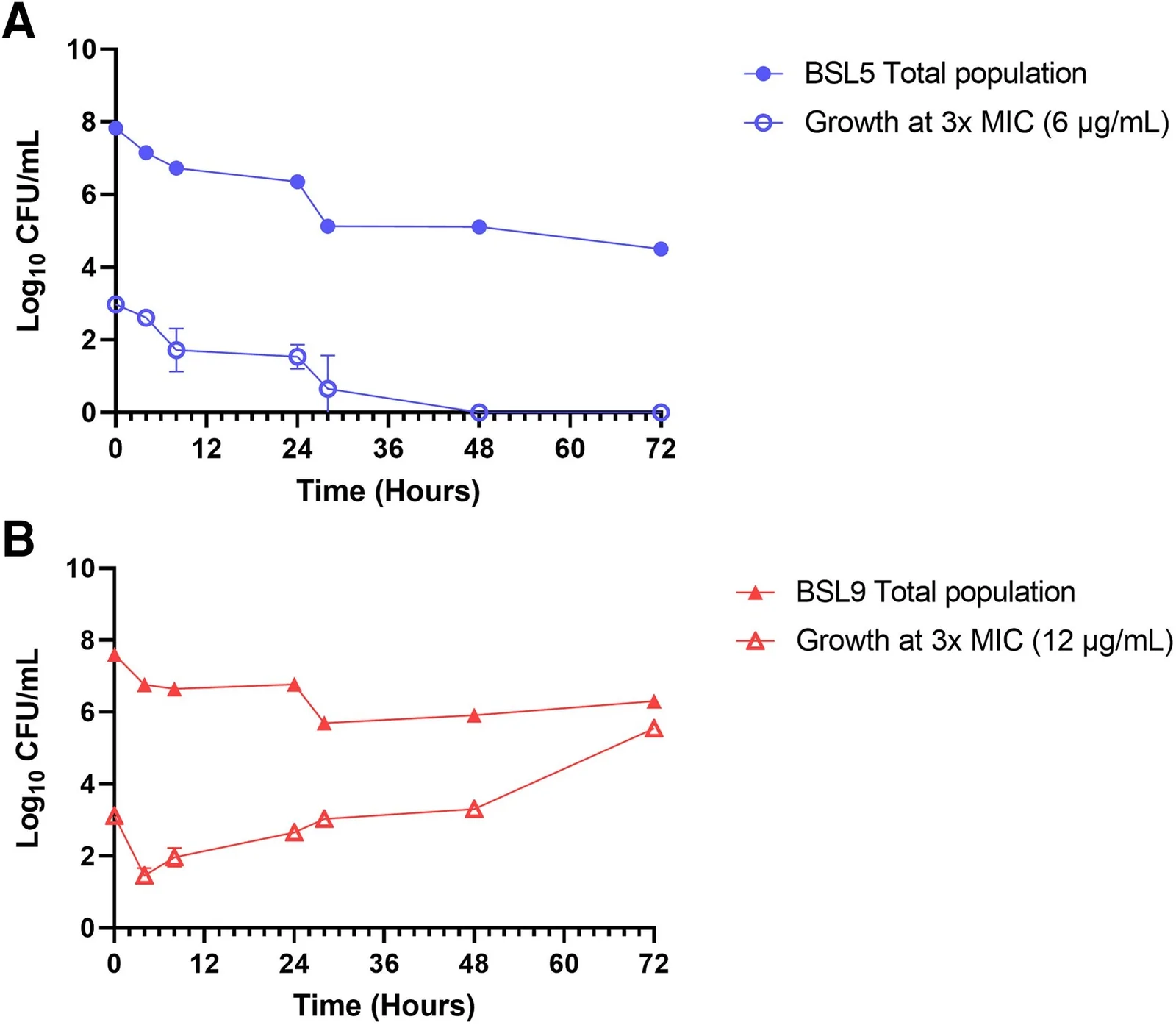
Impaired killing of the heteroresistant isolate BSL9 at human simulated cefiderocol exposures in a hollow fiber infection model. *A*, Susceptible strain BSL5 (cefiderocol MIC 2 µg/mL) and *B*, heteroresistant strain BSL9 (cefiderocol MIC 4 µg/mL) treated with human serum simulated exposure to cefiderocol 2 g over 3 h every 8 h. Log_10_ colony forming units (CFU)/mL of the total population are shown with filled shapes. CFU/mL recovered from resistance screening plates (3× MIC of parent isolate) are shown with open shapes.

## DISCUSSION

In this study we found that decreased susceptibility to cefiderocol was distributed across geographic locations and was present in diverse genetic backgrounds of *P. aeruginosa*. The proportion of heteroresistant isolates increased at higher MICs, with 8 of 9 total isolates at an MIC of 4 µg/mL being heteroresistant. It is notable that cefiderocol breakpoints are lower for both the United States Food and Drug Administration (susceptible ≤1 µg/mL) and EUCAST (susceptible ≤2 µg/mL), which would identify 39% (11/28) and 29% (8/28) of heteroresistant isolates from this cohort as nonsusceptible, respectively. Mean disk diffusion diameters were also significantly smaller for heteroresistant and nonsusceptible isolates, although the degree of overlap precludes establishing a simple zone diameter cutoff for definitive screening.

Since the POP cohort was enrolled prior to the clinical introduction of cefiderocol [23], and antimicrobial exposure data were not collected as a part of the study, we were not able to directly assess the clinical impact of decreased cefiderocol susceptibility on therapeutic outcomes with cefiderocol or associations with prior antimicrobial exposures. However, there are several important observations with potential clinical relevance. First, isolates with reduced cefiderocol susceptibility did not show differences in rates of colonization versus infection, suggesting that fitness defects from altered iron uptake or presence of resistance determinants may not manifest in a clinically meaningful difference in virulence among these isolates. Second, there were important geographic differences in the genomic features associated with cefiderocol resistance in this cohort. Acquired β-lactamases were more frequent in isolates outside the United States, while changes in AmpC and the PirRS pathway were associated with US isolates. Thus, strategies such as pairing cefiderocol with a β-lactamase inhibitor may overcome resistance in some regions, but may not be effective in parts of the world where altered uptake predominates [26]. Third, isolates with resistance to novel BL/BLIs, particularly ceftolozane/tazobactam and ceftazidime/avibactam, were associated with an increased probability of the isolate displaying reduced cefiderocol susceptibility. When placed in the context of the genomic results, where isolates with reduced cefiderocol susceptibility more commonly possess *bla*_NDM_, *bla*_VEB_, or mutations in *ampC* predicted to alter ceftolozane hydrolysis, there is the concern that the emergence of resistance to these BL/BLIs may compromise cefiderocol as an option for salvage therapy [26, 27]. Additionally, 6 isolates possessed large genomic deletions that included the genes encoding *piuA*, *piuC*, and *ampD*, among others. Similar deletions have been described in *P. aeruginosa* that developed resistance to ceftazidime-avibactam, highlighting the risk of emerging cross-resistance [34]. Further clinical data is needed to determine the risk of cefiderocol resistance after failing other anti-pseudomonal regimens and the optimal sequence of therapy for resistant *P. aeruginosa* infections.

Importantly, we utilized a human simulated dosing regimen in a hollow fiber model that supports the potential for heteroresistance to lead to therapeutic failure. Even with the free drug concentration above the isolate MIC for the entire dosing interval, cefiderocol was unable to suppress the emergence of resistance in the heteroresistant strain. Clinical scenarios where there may be reductions in cefiderocol exposure, such as penetration into epithelial lining fluid, or an uncontrolled source with a high burden of organisms could exacerbate the potential for the emergence of resistance. Further in vivo studies with heteroresistant isolates and defined mechanistic pathways are needed to delineate the risk of cefiderocol failure.

This study has several limitations. We utilized a prospective cohort from prior to the introduction of cefiderocol, thus we could not directly assess cefiderocol treatment outcomes. While we designed the sampling approach to capture the genetic diversity of TonB receptors, *ampC* alleles, and acquired β-lactamases of the cohort, and included closest related isolates without mutations as a control, the phenotypic cohort tested here may not reflect the full cohort. Since the strains from China were not included in the ARLG biorepository, we were unable to phenotypically characterize these isolates. Nonetheless, there were minimal differences between the selected isolates and the overall POP population (Supplementary Table 2) and no differences in the MIC distributions and resistance patterns of other novel BL/BLIs between the selected isolates and the overall cohort (Supplementary Table 3). This study included only CRPA, thus the higher rates of decreased susceptibility to cefiderocol we observed may be due to the multidrug-resistant nature of the isolates (the likely phenotype for which cefiderocol would be utilized). Whole-genome sequencing does not capture differences in gene expression, which may be important for differentiating isolates with resistant subpopulations, and our limited CNV analysis does not exclude this as a potential mechanism for heteroresistance on antibiotic exposure. In addition, we did not experimentally determine the impact of the observed mutations on cefiderocol MIC in an isogenic strain background; however, we and others have recently published data linking *pirR*, *fptA*, and *cpxS*, among other genes, to decreased cefiderocol susceptibility [13, 16]. Finally, while PAP assay is the gold standard for defining heteroresistance in vitro, the correlation between heteroresistance, AUC, and clinical therapeutic outcomes in *P. aeruginosa* remains to be established, although emerging data suggests this phenotype may impact clinical outcomes in bloodstream and urinary tract infections for other Gram-negative organisms [35, 36].

In summary, reduced cefiderocol susceptibility was present in isolates across geographic regions and genomic backgrounds in this large international cohort of carbapenem-resistant *P. aeruginosa.* High rates of cross-resistance to newer BL/BLIs were seen in isolates with reduced cefiderocol susceptibility and resistance emerged at human simulated cefiderocol exposures with a heteroresistant isolate tested in the hollow fiber infection model. This raises concerns with the use of cefiderocol as salvage therapy for CRPA infections with resistance to newer BL/BLIs, and additional research is needed to understand the implications of this phenotype in the clinical setting.

## Supplementary Material

jiag263_Supplementary_Data

## Supplementary Data

Supplementary materials are available at *The Journal of Infectious Diseases* online (http://jid.oxfordjournals.org/). Supplementary materials consist of data provided by the author that are published to benefit the reader. The posted materials are not copyedited. The contents of all supplementary data are the sole responsibility of the authors. Questions or messages regarding errors should be addressed to the author.
