# Supplementary material for: Genomic and Clinical Features Associated With Reduced Susceptibility to Cefiderocol and Cross-Resistance to β-Lactam/β-Lactamase Inhibitors in a Global Cohort of Pseudomonas aeruginosa
Source: J Infect Dis. 2026 May 15;234(4):e985–95. doi: 10.1093/infdis/jiag263 (PMC13668332; doi:10.1093/infdis/jiag263)
Supplement: Supplementary file 1 [file jiag263_supplementary_data.zip › Egge_FDC POP Supplement_Revised_FINAL.pdf]

<u>**Supplementary Methods and Data for:**</u>

Genomic and Clinical Features Associated with Reduced Susceptibility to Cefiderocol and Cross-resistance to β-lactam/β-lactamase Inhibitors in a Global Cohort of *Pseudomonas aeruginosa*

Stephanie L. Egge MD[1,2,#], Donghoon Kang PhD[1,2], Nazanin Pouya PharmD[3], Philip C. Thornton MS[1,2], Erika Flores PhD[1,2], Karim Clarke[1,2], Max W. Adelman MD[1,2,5], Cornelius L. Rosenbaum PhD[4], Rodrigo Baptista PhD[1,2,5], Cesar A. Arias MD[1,2,5], Vincent H. Tam PharmD[3], Michael J. Satlin MD[5], William R. Miller MD[1,2,5]

1. Division of Infectious Diseases, Houston Methodist Hospital, Houston, Texas, USA
2. Center for Infectious Diseases, Houston Methodist Research Institute, Houston, Texas, USA
3. Department of Pharmacy Practice and Translational Research, University of Houston College of Pharmacy, Houston, Texas, USA
4. Center of Health Data Science and Analytics, Houston Methodist, Houston, Texas, USA
5. Department of Medicine, Weill Cornell Medical College, New York, New York, USA

#Current Address: Department of Medicine, Division of Infectious Diseases, Oregon Health and Science University, 3181 SW Sam Jackson Park Road, Portland, OR 97239

Table of Contents

Supplementary Methods……..2
References……...4
Supplementary Figure 1……..6
Supplementary Figure 2……..7
Supplementary Figure 3……..8
Supplementary Figure 4……..9
Supplementary Figure 5……10
Supplementary Table 2……..11
Supplementary Table 3……..12

Supplementary Methods

Screening and genomic analysis of P. aeruginosa isolates from the Antibiotic Resistance Leadership Group (ARLG) Prospective Observational Pseudomonas (POP) Study.

The ARLG POP study enrolled 972 patients with cultures positive for carbapenem-resistant *P. aeruginosa* globally from December 2018 to November 2019, with index bacterial isolates (first eligible CRPA culture isolate per patient), susceptibility testing data, whole genome sequences, and de-identified clinical data maintained in a biorepository [1]. Antimicrobial exposure data were not collected as a part of the POP study. All 972 POP isolate genomes were assessed for mutations in TonB-dependent receptor genes that had been previously implicated in cefiderocol heteroresistance: *pirR, pirS, pirA, piuA/D, piuC*, as well as the *ampC* gene encoding the intrinsic Pseudomonas derived cephalosporinase (PDC) [2, 3]. Briefly, isolates had previously undergone DNA extraction using Wizard Genomic DNA purification kit (Promega) or DNeasy blood and tissue kit (Qiagen), libraries generated with Illumina Nextera XT DNA sample preparation kit (Illumina), and sequenced on an Illumina HiSeq 4000, NextSeq 2000, or MiSeq [1]. Draft genomes were assembled with SPAdes (version 3.15.4), annotated with PROKKA (version 1.14.6), and differences in nucleotide and protein amino acid sequence were determined using SNIPPY (version 4.6.0) with *P. aeruginosa* PAO1 as the reference [4]. Maximum Likelihood trees for each gene were constructed using RAxML-ng (version 1.2.0) to group the different gene alleles, and isolates were selected at random along with nearest phylogenetic neighbors with wild type TonB-dependent receptor genes to serve as a control population [5]. We identified 41 variants of *pirR*, 61 variants of *pirS*, 53 variants of *piuA*, 29 variants of *piuC*, and 47 variants of *ampC*. In addition, resistance determinants were identified using AMRFinderPlus (version 3.11.26) and unique isolates harboring genes encoding a diverse set of exogenous β-lactamases were also selected [6]. The resulting subset of isolates (n= 186, phenotypic cohort) encapsulated the permutations of TonB-dependent receptor gene mutations and β-lactamases contained within the POP cohort (**Supplemental Figure 1**). The identified bacterial strains and associated data were acquired from the ARLG isolate bank for further genomic and phenotypic characterization.

Maximum likelihood phylogenetic trees were constructed for the full POP cohort of 972 isolates, as well as the phenotypic cohort of 196 isolates. Briefly, core genomes of the isolates were identified using Roary, aligned with MAFFT (version 7.5.11), concatenated, then trees were constructed with RAxML using 1000 bootstrap resampling and visualized with iTol [7-11]. The genomes from the phenotypic cohort were then assessed for non-synonymous single nucleotide polymorphisms (SNPs), insertions, or deletions affecting genes implicated in changes in cefiderocol susceptibility using SNPEff (version 4.5) [12]. This included TonB-dependent receptors or their signaling pathways (*pirR, pirS, pirA, piuA, piuD, piuC, pfuA, femA, optE, fptA*), genes encoding the TonB complex (*tonB1, tonB2, exbB1, exbD1*), *ampC*, *ftsI* (encoding penicillin binding protein [PBP] 3), and *cpxS* (regulates expression of the MexAB-OprM and MuxABC-OpmB efflux pumps). These were further categorized into SNPs resulting in single amino acid changes, and probable loss of function mutations (defined as insertion, deletion, frameshift, or premature stop codons in the gene coding sequence). The identified *ampC* mutations were assessed for amino acid changes that are predicted to alter ceftolozane hydrolysis (F121L, G157D, ΔR212-L218, V213A, G216D P217L, E221K, E221G, A293P, ΔA293-L294, L294P, Q295R, numbers given reflect removal of the 26 amino acid signal peptide) [13, 14]. Of the original selection, a minority of strains (10 of 196 isolates) clustered on a distinct phylogenetic branch consistent with the proposed species *P. paraeruginosa* (type strain PA-7, average nucleotide identity (ANI) of ~93.5% as compared to *P. aeruginosa sensu stricto*) [15]. These strains were excluded from the analysis owing to the significant difference in ANI.

Bacterial strains and growth conditions

Bacterial stocks of the phenotypic cohort were stored in Brucella broth with 15% glycerol at -80° C. Isolates were cultured on Cetrimide agar (Thermo Scientific) at 37° C unless otherwise noted. Mueller Hinton (MH) II broth (BBL Becton Dickinson, FisherScientific) was iron depleted according to the protocol in Appendix H of the Clinical and Laboratory Standards Institute (CLSI) M100 document [16]. Iron chelation was performed using Chelex resin (Biorad Laboratories), with verification of iron levels below ≤ 0.03 mg/L using a colorimetric test (MQuant, MilliporeSigma). The strains *Escherichia coli* ATCC 25922 (cefiderocol MIC 0.06 µg/mL), *P. aeruginosa* PAO1 (cefiderocol MIC 0.5 µg/mL), and *P. aeruginosa* C1408 (cefiderocol MIC 16 µg/mL) served as control strains.

Antimicrobial susceptibility testing

Antimicrobial susceptibility testing for ceftolozane/tazobactam, ceftazidime-avibactam, and imipenem-relebactam was previously performed at the ARLG central laboratory [1]. Broth microdilution (BMD) testing for cefiderocol (Shionogi, Inc.) was performed using iron-depleted cation adjusted MH broth (ID-MH) based on CLSI guidelines [16]. All strains were grown overnight in ID-MH broth at 37° C with rotation to ensure maximal expression of TonB-dependent receptors and no cross contamination with iron-containing media. Testing was performed in triplicate on independent days. Modal minimum inhibitory concentrations (MICs) and MIC range are reported; if a strain had three different values on testing (e.g., MIC tested at 1, 2, and 4 on the three replicates) an additional two independent assays were performed to obtain a modal MIC. Kirby-Bauer disk diffusion testing was performed on MH agar (BBL Becton Dickinson, FisherScientific) using 30 µg cefiderocol disks (Hardy Diagnostics). Disk diameters were read at 24 hours and 48 hours, and were measured based on the clear zone of growth (i.e., no inner zone colonies) where applicable.

Assessment of heteroresistance by population analysis profile

Heteroresistance was assessed by population analysis profile (PAP) as previously described [2]. Briefly, MH agar (BD, FisherScientific) was supplemented with cefiderocol at concentrations of 0, 4, 8, and 16 µg/mL. All studies were performed using strains grown overnight in ID-MH adjusted to a controlled bacterial inoculum of 0.5 McFarland standard, corresponding to ~ $1X10^8$ colony forming units (CFU)/mL, in fresh ID-MH before serial dilution and plating. CFU counts were performed at 24 and 48 hours. Log-normalized changes in colony forming units at each antibiotic concentration were calculated based on the difference in value from the growth control. The trapezoidal method was used to calculate area under the curve (AUC) for each strain in Prism (GraphPad). Cut-offs for heteroresistance were assessed by both a -6 $\log_{10}$ change in CFU at the antimicrobial susceptibility breakpoint of 8 µg/mL, as well as AUC >80 (based on the 99.9% confidence interval of control strain PAO1) [2, 17]. Isolates that screened negative (i.e., a >6 $\log_{10}$ decrease in CFU/mL) on PAP and had a susceptible BMD MIC were assayed once and considered fully susceptible. Isolates that tested heteroresistant (by either $\log_{10}$-fold change or AUC criteria) or had a BMD MIC of ≥8 µg/mL had PAP assays repeated in triplicate on different days to confirm the phenotype.

Hollow fiber infection model

Two clinical *P. aeruginosa* isolates consecutively isolated from the same patient were examined (BSL5, BSL9) were evaluated [18]. Prior to the experiment, cefiderocol susceptibility of the isolates was determined using the broth dilution method in duplicate. Bacterial response to cefiderocol was evaluated in the hollow-fiber infection model (HFIM) using simulated serum exposures of 2 g administered over 3 hours every 8 hours. The target unbound Cmax for cefiderocol was 60 mg/L, with a target half-life of 2.5 hours. Serial samples were obtained in duplicates from the circulatory loop of the HFIM to verify the simulated drug exposures. Cefiderocol concentration in the samples were assayed using a validated LC-MS/MS method [19, 20]. Ceftazidime was used as the internal standard. Tryptic soy broth supplemented with deferiprone (15 µg/mL) was used as diluent [21].

A baseline inoculum of approximately 8 log CFU/mL was used. Changes in bacterial density were monitored for up to 72 hours through serial sampling. Two independent samples were collected at each timepoint from the bioreactor cartridge. These samples were plated quantitatively on Mueller–Hinton agar (MHA) plates, and viable colony counts were determined after 24 hours of incubation. To detect the emergence of resistance during the experiment, quantitative cultures were also undertaken using MHA plates supplemented with cefiderocol (at 3×MIC of the respective strain).

Statistical analysis

Differences in the median disk zone diameters between susceptible and heteroresistant or non-susceptible isolates were determined using Mann Whitney test using two sided P-value of <0.05 as the cutoff for significance. Clinical characteristics of the POP cohort were expressed as the median and range for continuous data, and frequency and percentage for categorical data. Descriptive statistics for the combined cefiderocol heteroresistant and non-susceptible population versus the cefiderocol susceptible population were calculated, with a significance threshold of P<0.05. Differences in continuous variables were determined using a two-sample t test with equal variance. Differences in categorical variables were determined using chi square test. Differences in the MIC distribution for ceftolozane-tazobactam, ceftazidime-avibactam, and imipenem-relebactam for the phenotypic cohort and the overall POP cohort were compared using chi square test.

Fisher's exact categorical analyses were used to assess associations between cefiderocol susceptibility phenotype and TBDR target gene (*pirRS, pirA, pirS, piuA/D, fptA, fiuA, fepA, optE, tonB1, tonB2, exbB1, exbD1), ampC, cpxS, ftsI*, and exogenous β-lactamase genes. Three-category analysis looking at overall differences in susceptible, heteroresistant, and non-susceptible isolates was performed for each gene of interest. Odds-ratio and associated 95% confidence intervals were calculated assessing odds of gene collective presence among cefiderocol heteroresistant plus non-susceptible versus susceptible strains.

Overall prevalence of resistance to ceftolozane-tazobactam, ceftazidime-avibactam, and imipenem-relebactam between cefiderocol susceptible and heteroresistant/non-susceptible isolates was compared using Chi square test. We determined odds of cefiderocol heteroresistance or non-susceptibility according to ceftolozane-tazobactam, ceftazidime-avibactam, and imipenem-relebactam resistance (as well as combinations of these drugs) using univariable logistic regression models. Logistic regression was also used to assess overall association of the cefiderocol heteroresistant or non-susceptible phenotype with total number of resistant β-lactam/β-lactamase inhibitors (BL/BLIs). We then constructed multivariable logistic regression models to assess independent association of each drug resistance with cefiderocol resistance. Data were analyzed with SAS Studio (SAS Institute, Cary, NC).

References

1. Reyes J, Komarow L, Chen L, et al. Global epidemiology and clinical outcomes of carbapenem-resistant Pseudomonas aeruginosa and associated carbapenemases (POP): a prospective cohort study. Lancet Microbe **2023**; 4:e159-e70.

2. Egge SL, Rizvi SA, Simar SR, et al. Cefiderocol heteroresistance associated with mutations in TonB-dependent receptor genes in Pseudomonas aeruginosa of clinical origin. Antimicrob Agents Chemother **2024**; 68:e0012724.

3. Streling AP, Al Obaidi MM, Lainhart WD, et al. Evolution of Cefiderocol Non-Susceptibility in Pseudomonas aeruginosa in a Patient Without Previous Exposure to the Antibiotic. Clin Infect Dis **2021**.

4. Bankevich A, Nurk S, Antipov D, et al. SPAdes: a new genome assembly algorithm and its applications to single-cell sequencing. J Comput Biol **2012**; 19:455-77.

5. Kozlov AM, Darriba D, Flouri T, Morel B, Stamatakis A. RAxML-NG: a fast, scalable and user-friendly tool for maximum likelihood phylogenetic inference. Bioinformatics **2019**; 35:4453-5.

6. Feldgarden M, Brover V, Gonzalez-Escalona N, et al. AMRFinderPlus and the Reference Gene Catalog facilitate examination of the genomic links among antimicrobial resistance, stress response, and virulence. Sci Rep **2021**; 11:12728.

7. Edgar RC. MUSCLE: multiple sequence alignment with high accuracy and high throughput. Nucleic Acids Res **2004**; 32:1792-7.

8. Letunic I, Bork P. Interactive Tree Of Life (iTOL) v4: recent updates and new developments. Nucleic Acids Res **2019**; 47:W256-W9.

9. Page AJ, Cummins CA, Hunt M, et al. Roary: rapid large-scale prokaryote pan genome analysis. Bioinformatics **2015**; 31:3691-3.

10. Stamatakis A. RAxML version 8: a tool for phylogenetic analysis and post-analysis of large phylogenies. Bioinformatics **2014**; 30:1312-3.

11. Katoh K, Standley DM. MAFFT multiple sequence alignment software version 7: improvements in performance and usability. Mol Biol Evol **2013**; 30:772-80.

12. Cingolani P, Platts A, Wang le L, et al. A program for annotating and predicting the effects of single nucleotide polymorphisms, SnpEff: SNPs in the genome of Drosophila melanogaster strain w1118; iso-2; iso-3. Fly (Austin) **2012**; 6:80-92.

13. Berrazeg M, Jeannot K, Ntsogo Enguene VY, et al. Mutations in beta-Lactamase AmpC Increase Resistance of Pseudomonas aeruginosa Isolates to Antipseudomonal Cephalosporins. Antimicrob Agents Chemother **2015**; 59:6248-55.

14. Gonzalez-Pinto L, Blanco-Martin T, Alonso-Garcia I, et al. Impact of transferable beta-lactamases and intrinsic AmpC amino acid substitutions on the activity of cefiderocol against wild-type and iron uptake-deficient mutants of Pseudomonas aeruginosa. J Antimicrob Chemother **2024**.

15. Rudra B, Duncan L, Shah AJ, Shah HN, Gupta RS. Phylogenomic and comparative genomic studies robustly demarcate two distinct clades of Pseudomonas aeruginosa strains: proposal to transfer the strains from an outlier clade to a novel species Pseudomonas paraeruginosa sp. nov. Int J Syst Evol Microbiol **2022**; 72.

16. Performance Standards for Antimicrobial Susceptibility Testing, 35th Edition. M-100. 34 ed: Clinical and Laboratory Standards Institute, **2025**.

17. Band VI, Hufnagel DA, Jaggavarapu S, et al. Antibiotic combinations that exploit heteroresistance to multiple drugs effectively control infection. Nat Microbiol **2019**; 4:1627-35.

18. Teran N, Egge SL, Phe K, Baptista RP, Tam VH, Miller WR. The emergence of cefiderocol resistance in Pseudomonas aeruginosa from a heteroresistant isolate during prolonged therapy. Antimicrob Agents Chemother **2024**; 68:e0100923.

19. Pouya N, Smith JE, Hudson CS, Teran NS, Tam VH. In vitro evaluation of using ceftazidime/avibactam against carbapenem-resistant Acinetobacter baumannii. J Glob Antimicrob Resist **2024**; 38:252-5.

20. Finch NA, Granillo A, Pouya N, Bhimraj A, Miller WR, Tam VH. Pharmacokinetics of cefiderocol in a patient with carbapenem-resistant Acinetobacter baumannii ventriculitis: A case report. Pharmacotherapy **2025**; 45:66-9.

21. Eales BM, Smith JE, Pouya N, Teran NS, Miller WR, Tam VH. Alternative iron-depleted media for cefiderocol susceptibility testing. Int J Antimicrob Agents **2024**; 64:107193.

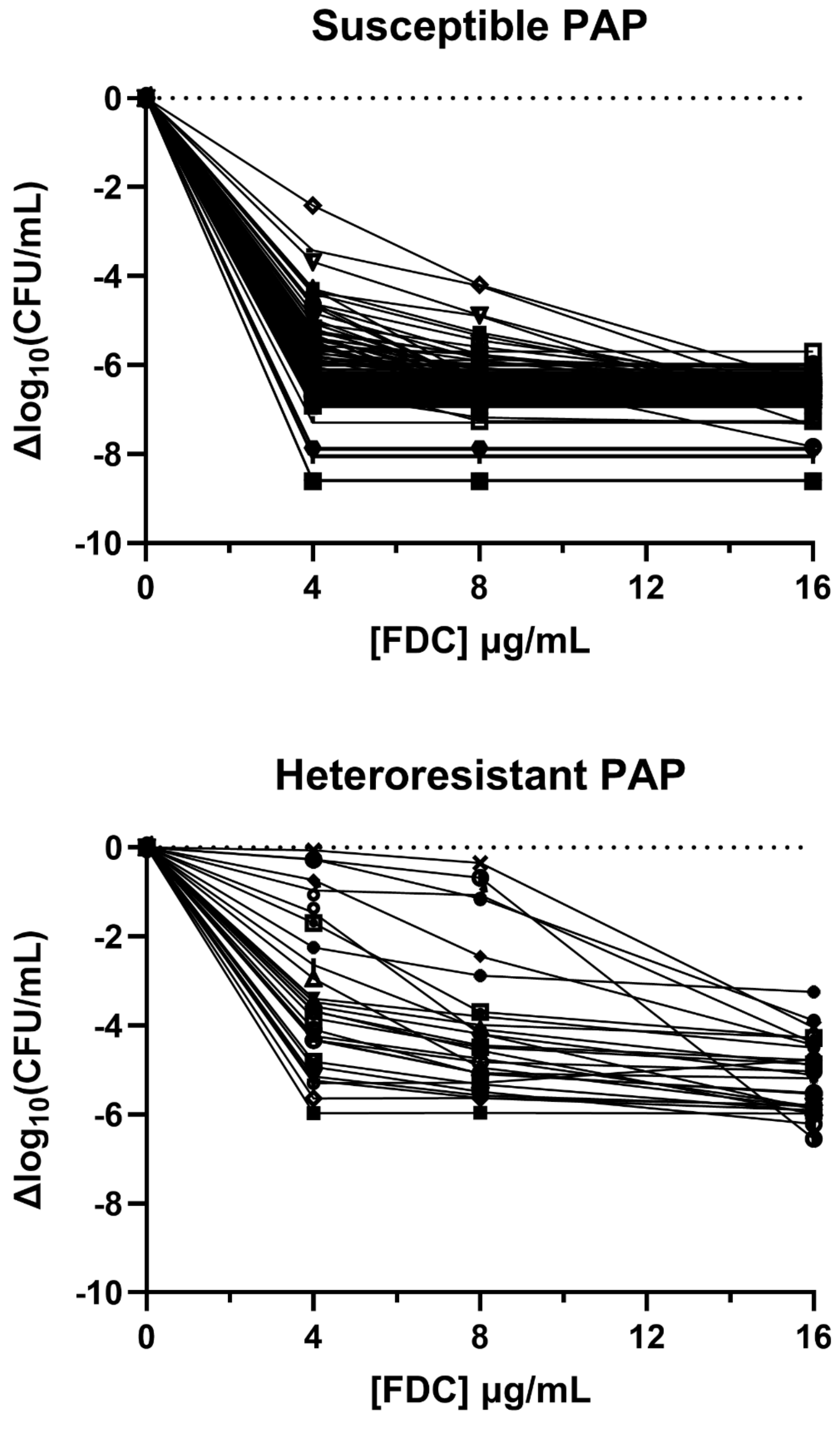

Supplemental Figure 1. Population analysis profile (PAP) curves for the phenotypic cohort. A total of 186 isolates were characterized phenotypically. Cefiderocol susceptible isolates (AUC ≤80) are shown in the top panel. Cefiderocol heteroresistant isolates (AUC >80) are shown in the bottom panel. Log_{10} change in CFU per mL from the initial inoculum is graphed on the y-axis, with cefiderocol concentration on the x-axis.

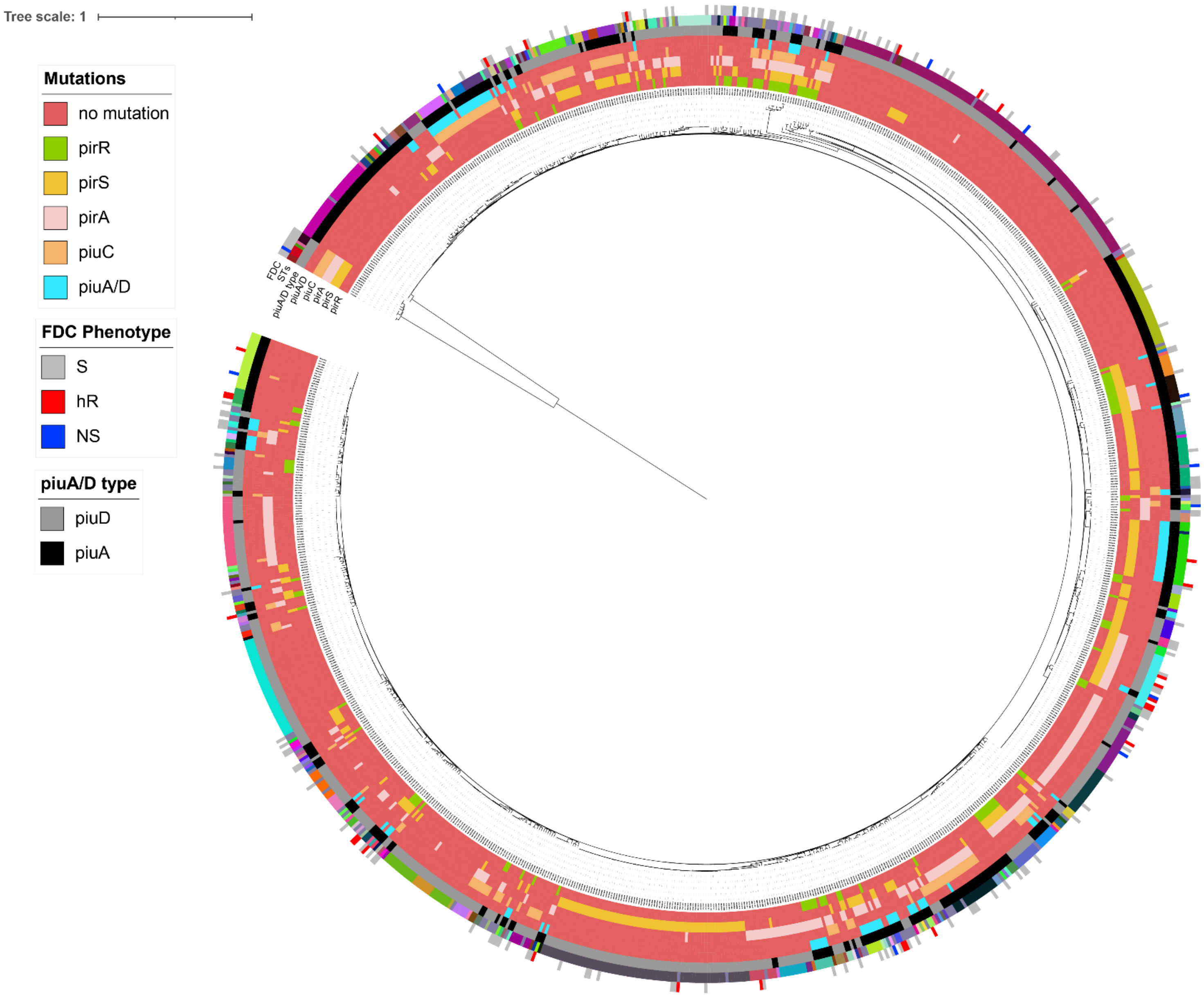

Supplemental Figure 2. Maximum likelihood phylogenetic tree of the POP cohort (n=972 isolates). The outermost ring indicates the isolates selected for the phenotypic cohort, grey bars are cefiderocol susceptible isolates, red bars are heteroresistant isolates, and blue bars are non-susceptible isolates. Isolates not tested are white. Moving inward, rings display sequence type, presence of *piuA* or *piuD* allele, and the presence of polymorphisms in genes from Ton-B dependent receptor systems as compared to the PAO1 reference.

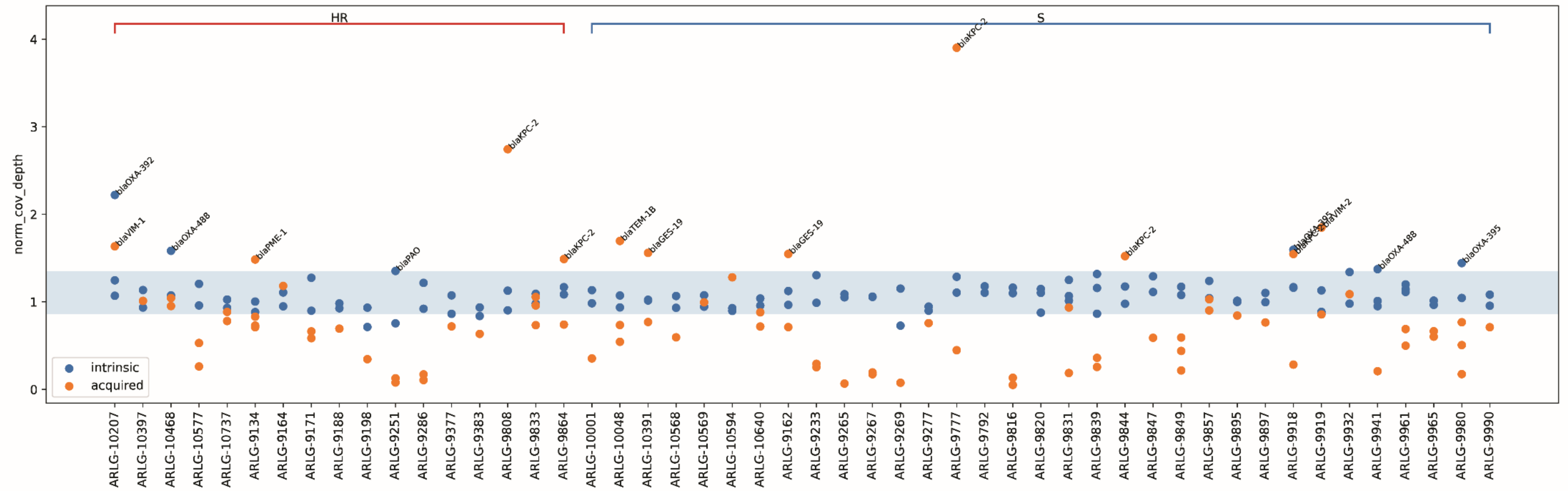

Supplemental Figure 3. Estimated copy number variation for isolates with acquired β-lactamase genes. Gene copy number variation was estimated from Illumina sequencing reads using CONVICT (https://github.com/wshropshire/convict). This tool estimates copy number from sequencing depth as compared to a set of housekeeping genes (the 7 genes from the *P. aeruginosa* MLST typing scheme). Acquired β-lactamase genes for each isolate are shown in orange, while the intrinsic *ampC* and *bla*$_{OXA-50}$ β-lactamase genes of *P. aeruginosa* are shown in blue. The blue shaded rectangle represents the 95% confidence interval for the intrinsic β-lactamase gene copy numbers. Individual strains are denoted on the x-axis, while normalized coverage depth of each gene (copy number variation) is shown on the y-axis.

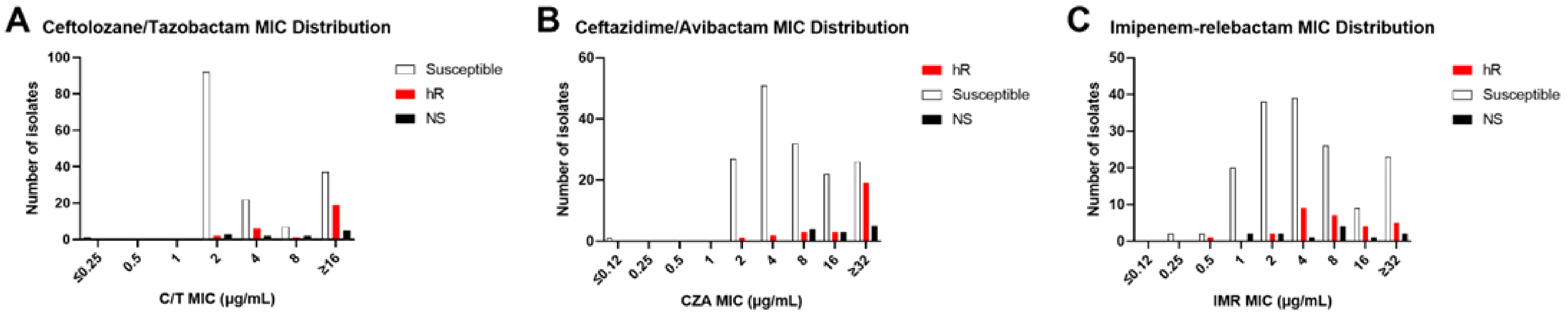

Supplemental Figure 4. Minimum inhibitory concentration (MIC) distributions for newer β-lactam/β-lactamase inhibitors according to cefiderocol susceptibility phenotype. hR: cefiderocol heteroresistant; NS: cefiderocol non-susceptible (CLSI designation intermediate or resistant) by broth microdilution; IMR: imipenem-relebactam; C/T: ceftolozane-tazobactam; CZA: ceftazidime-avibactam.

BSL5:

Cmax	65.39
V(L)	1.694
t1/2(h)	2.55
r^2	0.99

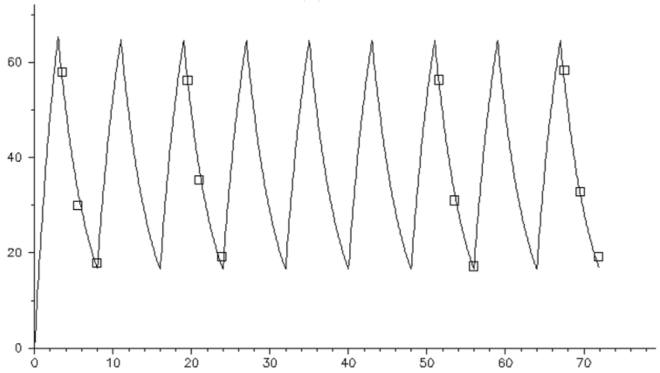

BSL9:

Cmax	60.68
V(L)	1.703
t1/2(h)	2.125
r^2	0.991

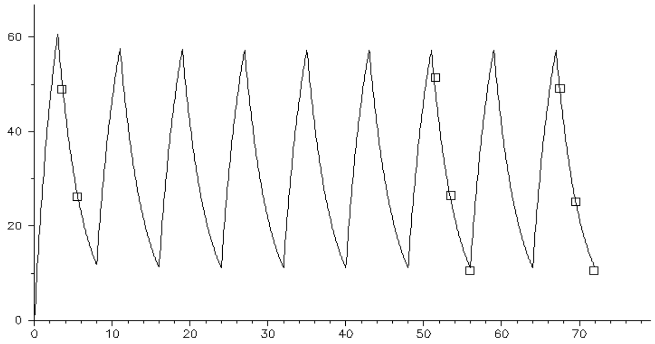

Supplemental Figure 5. Cefiderocol PK data for the hollow fiber infection model. Open symbols are observed concentrations and solid lines depict best-fit profiles. Cmax, peak concentration; FDC, cefiderocol; t1/2, half-life (in hours); V, volume (in liters).

Supplemental Table 2. Clinical features of patients from the phenotypic cohort compared to the remaining POP cohort.

	Phenotypic cohort, N=186[1]	Remaining cohort, N=786[1]	P-value[2]
Patient Demographics			
Age, at time of culture	59 (41-71)	63 (48-73)	0.051
Gender (Male : Female)	111 (60%) : 75 (40%)	510 (65%) : 276 (35%)	0.20
Geographic location			**< 0.01**
Asia	5 (2.7%)	207 (26.3%)	
Oceania	5 (2.7%)	10 (1.3%)	
Middle East	29 (16%)	62 (7.9%)	
North America	109 (59%)	418 (53%)	
South/Central America	38 (20%)	89 (11%)	
Patient Comorbidities			
Charlson Comorbidity Score	2 (0-3)	2 (1-4)	0.20
Chronic obstructive pulmonary disease	23 (13%)	131 (17%)	0.2
Cardiac disease	46 (25%)	225 (29%)	0.20
Cerebrovascular disease	32 (17%)	168 (22%)	0.20
Diabetes	54 (29%)	276 (35%)	0.13
Liver disease	11 (5.9%)	31 (4%)	0.20
Chronic kidney disease	31 (17%)	95 (12%)	0.10
Hemodialysis or Peritoneal Dialysis	21 (11%)	71 (9.1%)	0.30
Immunocompromised state	23 (13%)	100 (13%)	>0.9
Connective tissue disease	6 (3.3%)	28 (3.6%)	0.80
Hematopoietic stem cell transplant	2 (1.1%)	7 (0.9%)	0.70
Solid Organ Transplant	9 (4.9%)	29 (3.7%)	0.70
Heart	2 (1.1%)	4 (0.5%)	
Kidney	5 (2.7%)	14 (1.8%)	
Lung	2 (1.1%)	9 (1.1%)	
Liver	0 (0%)	2 (0.3%)	
Isolate Features			
Days from admission to culture	12 (1-31)	7 (1-27)	0.5
Patient location at first culture			0.13
Emergency department	25 (13%)	94 (12%)	
Medical ward	60 (32%)	240 (31%)	
Hematology/oncology ward	6 (3.2%)	19 (2.4%)	
Surgery ward	9 (4.8%)	84 (11%)	
Intensive care unit	76 (41%)	326 (41%)	
Other	10 (5.4%)	23 (2.9%)	
Source			**0.027**
Blood	15 (8.1%)	54 (6.9%)	
Respiratory	88 (47%)	435 (55%)	
Urine	38 (20%)	176 (22%)	
Wound	45 (24%)	121 (15%)	
Polymicrobial culture	61 (33%)	284 (36%)	0.40
Community v. Healthcare Onset	107 (58%) v. 79 (42%)	426 (54%) v. 360 (46%)	0.40
Infection (not colonization)	101 (54%)	480 (61%)	0.091

[1]*Median (Q1, Q3); n (%)*

[2]*Wilcoxon rank sum test; Pearson's Chi-squared test; Fisher's exact test*

Supplemental Table 3. Comparison of MIC distributions between the full POP cohort and isolates selected for FDC testing.

	Overall POP cohort		Phenotypic cohort		
	n (%)	95% CI	n (%)	95% CI	P-value[1]
**C/T MIC**					0.3
≤2	450 (56.2)	52.7-59.6	98 (49.2)	42.4-56.1	
4	104 (13.0)	10.8-15.5	30 (15.1)	10.8-20.7	
8	44 (5.5)	4.1-7.3	10 (5.0)	2.8-9.0	
≥16	203 (25.3)	22.5-28.5	61 (30.7)	24.7-37.4	
**CZA MIC**					0.15
≤0.5	7 (0.7)	0.4-1.5	0 (0)	0-1.9	
2	149 (15.3)	13.2-17.7	28 (14.1)	10.0-19.7	
4	258 (26.6)	23.9-29.4	53 (26.8)	21.1-33.3	
8	254 (26.2)	23.5-29.0	39 (19.7)	14.8-25.8	
16	120 (12.4)	10.4-14.6	28 (14.1)	10.0-19.7	
≥32	183 (18.8)	16.5-21.4	50 (25.3)	19.7-31.7	
**IMR MIC**					0.9
≤0.5	13 (1.6)	1.0-2.8	5 (2.5)	1.1-5.7	
1	79 (9.9)	8.0-12.2	22 (11.1)	7.4-16.2	
2	168 (21.0)	18.3-24.0	42 (21.1)	16.0-27.3	
4	211 (26.4)	23.5-29.6	49 (24.6)	19.2-31.1	
8	139 (17.4)	15.0-20.2	37 (18.6)	13.8-24.6	
16	47 (5.9)	4.5-7.7	14 (7.0)	4.2-11.5	
≥32	142 (17.8)	15.3-20.6	30 (15.1)	10.8-20.7	

[1]Chi-squared test
CI, confidence interval; C/T, ceftolozane/tazobactam; CZA, ceftazidime/avibactam; IMR, imipenem-relebactam.
